# Synthesis of Poly(methacrylic acid)-*block*-Polystyrene Diblock Copolymers at High Solid Contents via RAFT Emulsion Polymerization

**DOI:** 10.3390/polym13213675

**Published:** 2021-10-25

**Authors:** Iklima Oral, Larissa Grossmann, Elena Fedorenko, Jana Struck, Volker Abetz

**Affiliations:** 1 Institute of Physical Chemistry, Universität Hamburg, Grindelallee 117, 20146 Hamburg, Germany; iklima.oral@chemie.uni-hamburg.de (I.O.); larissa.grossmann@studium.uni-hamburg.de (L.G.); elena.fedorenko@chemie.uni-hamburg.de (E.F.); jana.struck@chemie.uni-hamburg.de (J.S.); 2 Helmholtz-Zentrum Hereon, Institute of Membrane Research, Max-Planck-Straße 1, 21502 Geesthacht, Germany

**Keywords:** reversible-addition-fragmentation chain transfer, high solid content, polymerization-induced self-assembly, emulsion polymerization, polystyrene, poly(methacrylic acid), *block* copolymers

## Abstract

The combination of polymerization–induced self-assembly (PISA) and reversible–addition fragmentation chain transfer (RAFT) emulsion polymerization offers a powerful technique to synthesize diblock copolymers and polymeric nanoparticles in a controlled manner. The RAFT emulsion diblock copolymerization of styrene and methacrylic acid (MAA) by using a trithiocarbonate as surfactant and RAFT agent was investigated. The Z-group of the RAFT agent was modified with a propyl-, butyl- and dodecyl- sidechain, increasing the hydrophobicity of the RAFT agent to offer well-controlled polymerization of poly(methacrylic acid)-*block*-polystyrene (PMAA-*b*-PS) diblock copolymers at high solid contents between 30–50 wt% in water. The kinetic data of the PMAA homopolymerization with the three different RAFT agents for various solvents was investigated as well as the RAFT emulsion polymerization of the diblock copolymers in pure water. While the polymerization of PMAA-*b*-PS with a propyl terminus as a Z-group suffered from slow polymerization rates at solid contents above 30 wt%, the polymerization with a dodecyl sidechain as a Z-group led to full conversion within 2 h, narrow molar mass distributions and all that at a remarkable solid content of up to 50 wt%.

## 1. Introduction

The advent of controlled radical polymerization (CRP) gave rise to a wide variety of well-defined homopolymers and copolymers [1,2,3,4]. RAFT is based on degenerative chain transfer, thus, exhibiting quasi-living characteristics while benefiting from the advantages of a radical process. Those advantages include high tolerance towards various functionalities and straightforward feasibility, allowing for the polymerization of a plethora of monomers [5]. The formulation is similar to a free-radical polymerization with the difference that it utilizes a dithioester or any of its derivatives as chain transfer agent (CTA) to control the active radical concentration and hence enabling uncomplicated tailoring of the average molecular weight and dispersity (*M*_w_*/M*_n_) of the polymer. Conventional radical polymerization does not facilitate control, i.e., customized molecular weight distributions, polymer compositions (when it comes to block-like or gradient structures) and polymer architectures. RAFT polymerization can be conducted in a range of solvents [6], including protic solvents such as water [7,8] and lower alcohols such as methanol [9] and ethanol [10], non-polar solvents, e.g., *n*-alkanes [6] and more exotic media such as ionic liquids [9,11]. It can be performed in bulk, solution or dispersion (with emulsion being a subclass of it) [12,13,14]. The combination of RAFT polymerization and aqueous emulsion polymerization can be employed with a wide range of hydrophobic monomers, additionally enabling polymerizations up to a high solid content while allowing high molecular weights and still maintaining low viscosities and an excellent heat transfer [15,16,17]. Furthermore, this combination offers a potential surfactant-free route for the efficient synthesis of nanosized latexes in water. Hawkett et al. [18,19] established the first successful ab initio RAFT emulsion polymerization using a poly(acrylic acid) (PAA) macro-CTA, which was chain extended in a emulsion polymerization with *n*-butyl acrylate to form stable latex particles. Charleux et al. established RAFT emulsion polymerization protocols in a series of innovative studies [20,21]. Different hydrophilic stabilizer blocks (prepared from acrylic acid, MAA and acrylamide), hydrophobic core forming blocks (containing *n*-butyl acrylate, styrene, methyl methacrylate, and benzyl methacrylate) and RAFT agents (both trithiocarbonates and dithiobenzoates) were developed [14,20,21,22,23,24,25,26]. Other parameters such as pH were studied in detail, the reaction conditions were optimized to provide high monomer conversion within short reaction times, narrow size distributions and good control over the molecular weight [20,27]. In this work, PMAA-*b*-PS diblock copolymers were synthesized via RAFT aqueous emulsion polymerization using MAA as water soluble monomer and styrene as the water-immiscible monomer to produce an amphiphilic diblock copolymer in situ. This approach leads to a PISA and can produce diblock copolymer nanoparticles in the form of either spheres, worms, or vesicles. The general procedure of PISA via RAFT polymerization is the chain extension of a soluble homopolymer by a second monomer in a suitable solvent (as it is in this case a non-solvent for the extended polymer block). During the polymerization, the growing second block becomes more and more insoluble. Eventually, in situ self-assembly occurs and forms diblock copolymer nanoparticles.

The first PMAA block is synthesized via RAFT solution polymerization and forms the stabilizer block, while the second block is the core forming block and is synthesized via RAFT emulsion polymerization afterwards. The PMAA block as stabilizer in the formation of PMAA-*b*-PS block copolymers was deeply investigated by the group of Choe et al. [28]. PMAA-*b*-PS is an attractive block copolymer since the monomers are available on large scale, cheap and with the carboxylic acid within the polymer, it offers a variety of post-modification possibilities [29]. The latter is of particular interest as it allows different properties to be incorporated into the polymer system without the need for complex polymerization techniques.

**Figure 1:**
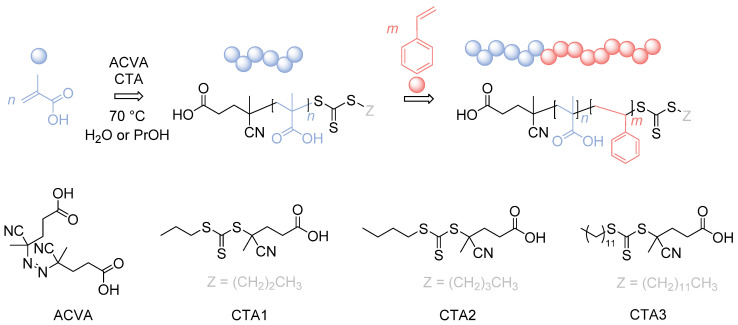
Reaction overview of the RAFT polymerization of PMAA-*b*-PS diblock copolymers using three different CTAs. CTA1: 4-Cyano-4-thioylthiopropylsulfanyl pentanoic acid (CPP), CTA2: 4-Cyano-4-thioylthiobutylsulfanyl pentanoic acid (CBP), CTA3: 4-Cyano-4-thioylthiododecylsulfanyl pentanoic acid (CDTPA).

In here, we will present a detailed study of the synthesis of PMAA-*b*-PS diblock copolymers at high solid concentrations by choosing three different CTAs, making this polymer even more attractive for industrial applications (Figure 1).

## 2. Materials and Methods

### 2.1. Materials

The 1-propanethiol (99%, Sigma-Aldrich, Schnelldorf, Germany), 1-butanethiol (>99%, Sigma-Aldrich, Schnelldorf, Germany), 4-cyano-4-thioylthiododecylsulfanyl pentanoic acid (CDTPA) (97%, abcr, Karlsruhe, Germany), MAA (>99%, Merck, Darmstadt, Germany), potassium hydroxide (KOH) (85%, Merck, Darmstadt, Germany), carbon disulfide (CS_2_) (99%, Merck, Darmstadt, Germany), *p*-tosyl chloride (98%, Merck, Darmstadt, Germany), 4,4′-azobis(4-cyanovaleric acid) (ACVA) (>98%, Sigma-Aldrich, Schnelldorf, Germany), 1,4-dioxane (99%, Grüssing, Germany), ethanol (99%, VWR Chemicals, Darmstadt, Germany), 1-propanol (PrOH) (99%, Grüssing, Filsum, Germany), *N*,*N*-dimethylformamide (DMF) (99%, VWR Chemicals, Darmstadt, Germany), tetrahydrofuran (THF) (>99%, VWR Chemicals, Darmstadt, Germany), styrene (99%, Grüssing, Filsum, Germany), dichloromethane (DCM) (>99%, Acros, Schwerte, Germany), acetone (>99%, Merck, Darmstadt, Germany), *n*-hexane (95%, VWR Chemicals, Darmstadt, Germany), chloroform-*d*_1_ (>99%, Euriso-Top, Saarbrücken, Germany), THF-*d*_8_ (>99%, Euriso-Top), dimethylsulfoxide-*d*_6_ (>99%, Germany), deuterium oxide (D_2_O) (>99%, Euriso-Top, Saarbrücken, Germany), trifluoroacetic acid (TFA) (99%, TCI Chemicals, Eschborn, Germany), NaHCO_3_ (>99%, Grüssing, Filsum, Germany), Mg_2_SO_4_ (>99%, Grüssing, Filsum, Germany), and activated basic aluminum oxide (>99%, grain size between 0.063–0.200 mm, Merck, Darmstadt, Germany) were used as received without further purification unless noted otherwise. Styrene was purified by filtration through basic activated aluminum oxide to remove the inhibitor. Further, 4-Cyano-4-(propylsulfanylthiocarbonyl)sulfanyl pentanoic acid (CPP) and 4-Cyano-4-(butylsulfanylthiocarbonyl)sulfanyl pentanoic acid (CBP) were synthesized according to the literature (see Section 2.3 and Section 2.4). Deionized water was purified with a Milli-Q^®^ integral water purification system. The subscripts in the designation of the polymers represent the fraction of the respective block in wt% and the superscript represents the overall molecular weight in kDa.

### 2.2. General Methods

^1^H NMR and ^13^C NMR spectra were recorded at ambient temperature using a 300 or 400 MHz Bruker AVANCE II spectrometer in either CDCl_3_-*d*_1_, D_2_O or THF-*d*_8_. The residual signals at *δ* = 7.26 for CDCl_3_-*d*_1_, *δ* = 4.80 ppm for D_2_O and *δ* = 3.58 ppm for THF-*d*_8_ were used as an internal standard for the chemical shifts.

Molecular weight distributions of the PMAA homopolymers were measured on a PSS^®^ Agilent Technologies 1260 Infinity systems utilizing a SURPREMA^®^ column system consisting of a precolumn (8 mm × 50 mm, particle size: 5 µm) and three analytical columns (Column 1: 8 mm × 300 mm, particle size: 5 µm, mesh size: 1000 Å; Column 2: 8 mm × 300 mm, particle size: 5 µm, mesh size: 1000 Å; Column 3: 8 mm × 300 mm, particle size: 5 µm, mesh size: 30 Å). The system was operating with a flow rate of 1.0 mL·min^−1^ at 50 °C and the injection volume of the polymer solution (*c* = 1 g·L^−1^) was 100 μL. As water was used as an eluent, calibration was achieved using a series of near-monodisperse poly(ethylene oxide) (PEO) or PAA standards. Molecular weight distributions of the PMAA-*b*-PS diblock copolymer were obtained using THF SEC. Polymer solutions were prepared in THF containing toluene as internal reference. The SEC-set-up comprised an Agilent 1260 Infinity series degasser and pump, a combination of three PSS SDV^®^ columns (100 Å, 1000 Å, 10,000 Å) connected in series to both, a refractive index and a UV detector. The system was operating at a flow rate of 1.0 mL·min^−1^ at 30 °C and the injection volume of the polymer solution (*c* = 1 g·L^−1^) was 100 μL. Calibration was achieved using a series of near-monodisperse polystyrene standards for diblock copolymers. All diblock copolymers were modified via Steglich esterification [30] using methanol as methylation agent to avoid COOH group interactions with SEC columns [31]. To avoid post modification of the polymer, polymer solutions were also prepared in THF with 5 wt% TFA. This ensures that the acid groups do not interact with the PSS SDV^®^ columns of the THF SEC.

Dynamic light scattering (DLS) studies were performed using an ALV/CGS-3 Compact Goniometer system at 25 °C at a scattering angle of 90° with an equilibration time of 120 s. Diblock copolymer solutions were diluted in 0.01 wt% prior to light scattering studies. The intensity-average diameter, derived count rate (CR) and polydispersity (PDI) of the diblock copolymer particles were calculated by the cumulants method. Data were averaged over 3 runs. It should be noted that DLS reports intensity-average diameters and implicitly assumes a spherical morphology.

TEM images were taken with an Eagle™ 4k HS 200 kV camera on a FEI™ Tecnai™ G2 Spirit TWIN instrument in bright field mode, operating at an accelerating voltage of 120 kV. Images were processed with TEM imaging and Analysis Offline 4.7 SO3 (FEI™) software and ImageJ 1.53. Samples were prepared on carbon coated copper grids. Polymer dispersions (*c* = 1 mg mL^−1^) were drop cast onto the TEM grid before excess of solution was blotted with filter paper.

The high-performance liquid chromatography mass spectrometry (HPLC MS) experiments were performed using an Agilent 1200 HPLC instrument and an Agilent 6224 time of flight (ToF) sensor. The sample solution was delivered to the electron spray ionization (ESI) source by a syringe pump at a flow rate of 0.4 mL min^−1^ and an injection volume of 1.0 µL.

### 2.3. Synthesis of 4-Cyano-4-Thioylthiopropylsulfanyl Pentanoic Acid (CPP)

CPP RAFT agent was synthesized via a modified procedure adapted from Xu et al. [32]. The 1-Propanethiol (5.0 mL, 0.055 mmol) was added dropwise to a solution of KOH (3.9 g, 0.069 mmol) in 20 mL of water under nitrogen atmosphere. CS_2_ (3.3 mL, 0.055 mmol) was added in one portion to the resulting reaction mixture and vigorously stirred for 30 min at room temperature. Afterwards, the reaction mixture was cooled to −5 °C and a solution of *p*-tosyl chloride (5.3 g, 0.028 mmol) in distilled acetone (28 mL) was added in portions over 20 min. After stirring an additional 2 h at −5 °C, any acetone residue was removed under reduced pressure. The crude product was extracted with DCM (100 mL) and the organic layer was washed three times with deionized water (50 mL). The resulting solution was dried over anhydrous magnesium sulfate, filtrated, and evaporated to dryness to yield bis(propylsulfanylthiocarbonyl) disulfide as a red oil (7.24 g, 87%). In a second step, ACVA (8.89 g, 31.7 mmol) and bis(propylsulfanylthiocarbonyl) disulfide (4.80 g, 15.7 mmol) were dissolved in ethyl acetate (120 mL) and heated under reflux at 85 °C for 24 h. After removal of the volatiles in vacuo, the crude product was purified by column chromatography on silica gel with a mobile phase of *n*-hexane/ethyl acetate (3:1 *v*/*v*). The product was obtained as an orange powder (4.84 g, 55%). ^1^H NMR (400 MHz, CDCl_3_,*δ*): 3.31 (t, ^3^*J*(H,H) = 6 Hz, 2H, –S–CH_2_–CH_2_–CH_3_), 2.72–2.40 (m, 4H, COOH–CH_2_–CH_2_–C–), 1.87 (s, 3H, –C–CH_3_), 1.73 (m, 2H, –S–CH_2_–CH_2_–), 0.96 (t, ^3^*J*(H,H) = 6 Hz, 3H, –CH_2_-CH_3_); ^13^C NMR (400 MHz, CDCl_3_, *δ*): 220 (C=S), 178 (–COOH), 119 (–CN), 46.0 (C^4^–CN), 39.2 (–S–CH_2_–), 29.2 (CN–C^4^–CH_2_–), 24.8 (COOH–CH_2_–), 22.5 (S–CH_2_–CH_2_–), 21.3 (CN–C–CH_3_), 13.5 (–S–CH_2_–CH_2_–CH_3_); HRMS (ESI) *m*/*z*: [M+H]^+^ calcd. for C_10_H_15_N_3_O_2_S_3_, 278.0338; found, 278.0342.

### 2.4. Synthesis of 4-Cyano-4-Thioylthiobutylsulfanyl Pentanoic Acid (CBP)

CBP RAFT agent was synthesized via a modified procedure adapted from Xu et al. [32] 1-Butanethiol (5.26 g, 58.3 mmol) was added dropwise to a solution of KOH (3.86 g, 68.8 mmol) in 25 mL of water under nitrogen atmosphere. CS_2_ (4.29 g, 28.5 mmol) was added in one portion to the resulting reaction mixture and vigorously stirred for 30 min at room temperature. Afterwards, the reaction mixture was cooled to −5 °C and a solution of *p*-tosyl chloride (5.24 g, 27.5 mmol) in distilled acetone (35 mL) was added in portions over 20 min. After stirring an additional 2 h at −5 °C, any acetone residue was removed under reduced pressure. The crude product was extracted with DCM (100 mL) and the organic layer was washed three times with deionized water (50 mL). The resulting solution was dried over anhydrous magnesium sulfate, filtrated, and evaporated to dryness to yield bis(butylulfanylthiocarbonyl) disulfide as a red oil (7.96 g, 82%). In a second step, ACVA (2.34 g, 8.35 mmol) and bis(butylsulfanylthiocarbonyl) disulfide (1.41 g, 4.26 mmol) were dissolved in ethyl acetate (30 mL) and heated under reflux at 85 °C for 24 h. After removal of the volatiles in vacuo, the crude product was purified by column chromatography on silica gel with a mobile phase of petrol ether/ethyl acetate (3:2 *v*/*v*). The product was obtained as an orange powder (2.14 g, 86%). ^1^H NMR (300 MHz, CDCl_3_, *δ*): 3.39–3.28 (m, 2H, –S–CH_2_–), 2.74–2.63 (m, 2H, C^4^–CH_2_–), 2.62–2.31 (m, 2H, –CH_2_–COOH), 1.88 (s, 3H, –C^4^–CH_3_), 1.68 (m, 2H, –CH_2_–CH_2_–CH_3_), 1.42 (m, 2H, –CH_2_–CH_3_), 0.94 (t, ^3^*J*(H,H) = 7.3 Hz, 3H, –CH_3_). ^13^C NMR (400 MHz, CDCl_3_, δ): 217 (C=S), 177 (–COOH), 119 (–CN), 46.6 (–C^4^–CN), 37.2 (–C^4^–CH_2_–), 33.9 (–S–CH_2_–), 31,3 (–S–CH_2_–CH_2_–), 29.9 (COOH–CH_2_–), 25.3 (–C^4^–CH3), 22.5 (–S–CH_2_–CH_2_–CH_2_–), 14.0 (–CH_2_–CH_3_).

### 2.5. Synthesis of PMAA with CPP or CBP

A typical polymerization of PMAA with CPP as CTA adapted from Chaduc et al. was as follows [21]. MAA (3.6 g, 0.042 mol), CPP (0.097 g, 3.5 × 10^−4^ mol), ACVA (0.0098 g, 3.5 × 10^−5^ mol), water (15 mL) and DMF (0.54 mL, 7.0 × 10^−3^ mol) were added to the reaction flask and purged with nitrogen in an ice bath for 30 min. The sealed reaction flask was immersed into a preheated oil bath for 6 h at 80 °C. The reaction was quenched by exposure to air. Water was removed under reduced pressure, the residual oil re-dissolved in ethanol and precipitated into excess of diethyl ether. Any residual diethyl ether, water, or ethanol was removed by drying the precipitate in vacuo at room temperature for 24 h. The conversion was determined by ^1^H NMR spectroscopy in D_2_O.

### 2.6. Synthesis of PMAA with CDTPA

A typical polymerization of PMAA with CDTPA as CTA modified from Chaduc et al. was as follows [21]. MAA (2.4 g, 0.028 mol), CDTPA (0.093 g, 2.3 × 10^−4^ mol), ACVA (0.012 g, 4.6 × 10^−5^ mol), ethanol (15 mL) and DMF (0.35 mL, 4.6 × 10^−3^ mol) were added to the reaction flask and purged with nitrogen in an ice bath for 30 min. The sealed reaction flask was immersed into a preheated oil bath for 6 h at 80 °C. The reaction was quenched by exposure to air. The polymer solution was precipitated into excess of diethyl ether. Any residual diethyl ether or ethanol was removed by drying the precipitate in *vacuo* at room temperature for 24 h. The conversion was determined by ^1^H NMR spectroscopy in DMSO-*d*_6_.

### 2.7. One-Pot Synthesis of PMAA-b-PS with CPP or CBP

A typical polymerization of PMAA-*b*-PS with PMAA-CPP as macro-CTA was adapted from Chaduc et al. as follows [21]. MAA (3.4 g, 0.040 mol), CPP (0.055 g, 2.0 × 10^−4^ mol), ACVA (0.0056 g, 2.0 × 10^−5^ mol), 14 mL water and DMF (0.48 g, 6.7 × 10^−3^ mol) were added to the reactions flask and were purged with nitrogen in an ice bath for 30 min. The sealed reaction flask was immersed into a preheated oil bath for 6 h at 80 °C to reach full conversion. The reaction was quenched by exposure to air. A sample was taken for ^1^H NMR spectroscopy in D_2_O and SEC measurements in THF. A solution of styrene (8.3 g, 0.080 mol), ACVA (0.011 g, 4.0 × 10^−5^ mol), and NaHCO_3_ (0.012 g, 1.4 × 10^−5^ mol) in water (32 mL) was added to the reaction mixture, degassed with nitrogen for 30 min and immersed into a preheated oil bath for 2 h at 80 °C. After cooling to room temperature, water was removed under reduced pressure, the crude product re-dissolved in THF and precipitated into excess *n*-hexane. Any residual solvent was removed by drying the precipitate in vacuo at room temperature for 24 h. The conversion was determined by ^1^H NMR spectroscopy in THF-*d*_8_.

### 2.8. Synthesis of PMAA-b-PS with CDTPA

A typical polymerization of PMAA-*b*-PS with PMAA-CDTPA as macro-CTA was adapted from Chaduc et al. as follows [21]. A solution of styrene (1.35 g, 0.013 mol), ACVA (0.0036 g, 1.3 × 10^−5^ mol), and NaHCO_3_ (0.0038 g, 4.6 × 10^−5^ mol) and PMAA_120_-CDTPA (0,32 g, 6.5 × 10^−5^ mol) in water (2.5 mL) was added to the reaction flask, degassed with nitrogen for 30 min and immersed into a preheated oil bath for 2 h at 80 °C. After cooling to room temperature, water was removed under reduced pressure, the crude product re-dissolved in THF and precipitated into excess *n*-hexane. Any residual solvent was removed by drying the precipitate in vacuo at room temperature for 24 h. The conversion was determined by ^1^H NMR spectroscopy in THF-*d*_8_.

### 2.9. Determination of the Molecular Weight

The theoretical molecular weights of all polymers are calculated from the following Equation (1):

(1) $$M_{n,\mathrm{th}}=\frac{{\left[M\right]}_{0}\cdot p\cdot M_{M}}{{\left[\mathrm{CTA}\right]}_{0}}+M_{\mathrm{CTA}}$$

where [M]_0_ and [CTA]_0_ are the initial concentrations of monomer and chain transfer agent, *p* the monomer conversion and *M*_M_ and *M*_CTA_ are the molar masses of monomer and chain transfer agent. It follows that the molar mass increases linearly with conversion, assuming that all CTAs have reacted. The degree of polymerization (DP) is defined by the ratio of monomer to RAFT agent since most polymer chains are created from initiation by the R group of the RAFT agent [5,33].

## 3. Results and Discussion

### 3.1. Kinetics of the Homopolymerization of PMAA

The synthesis of PMAA-*b*-PS diblock polymers was performed according to the previous work of Chaduc et al. where the CTA1 was used and different pH-values, solvents, concentrations and reaction times were studied [34]. CTAs with a trithiocarbonate group were proven to be less prone to hydrolysis [35]. A carboxylic acid group was chosen as the R group of the RAFT agent, since it is similar to the structure of MAA, which will be polymerized as the hydrophilic block (see Figure 1). The R group was chosen to be a tertiary radical leaving group which is stable enough to allow both monomer addition and the cleavage process of the pre-equilibrium step of the RAFT mechanism. In here, we used three different CTAs with a trithiocarbonate group containing a *n*-propyl-, *n*-butyl- and *n*-dodecyl- terminus as a Z group to adjust the hydrophilicity of the CTA so that a good balance between the solution and emulsion RAFT polymerization in terms of solubility, reaction time, and latex stability is achieved.

**Figure 2:**
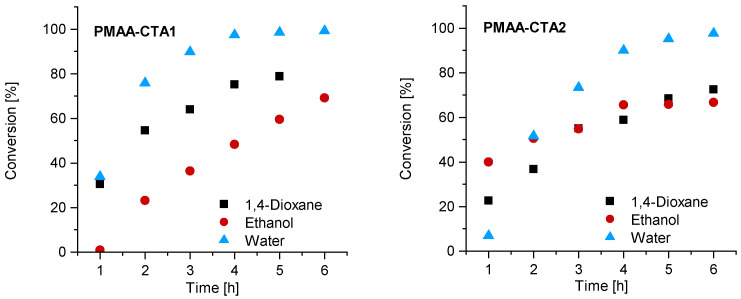
Reaction time versus conversion of the RAFT polymerization of PMAA with CTA1 (**left**) and CTA2 (**right**) with a molar ratio of [MAA]/[CTA] = 120 and [CTA]/[ACVA] = 10.

The first series of MAA RAFT solution polymerizations was conducted with CTA1 in water, 1,4-dioxane and ethanol. The CTA1 showed a high effectivity in RAFT polymerizations of methacrylate monomers leading to properly controlled living chains [32]. The polymerization was conducted at 70° C. The other reaction conditions such as the initial concentration of MAA, initiator and RAFT agent were kept constant. The polymerization of MAA with CTA1 in ethanol led to an inhibition period of 1 h, while the polymerization in 1,4-dioxane and water led to measurable monomer consumption already after 1 h. Almost full conversion in water was observed in less than 4 h, while the polymerization in 1,4-dioxane led to 75% (4 h) and in ethanol to only 48% conversion after 5 h (Figure 2). Similar observations were found in the publication of Chaduc et al. In this publication, methanol was used instead of ethanol, however, comparable results were obtained with a conversion of 59% after 7 h [34].

**Figure 3:**
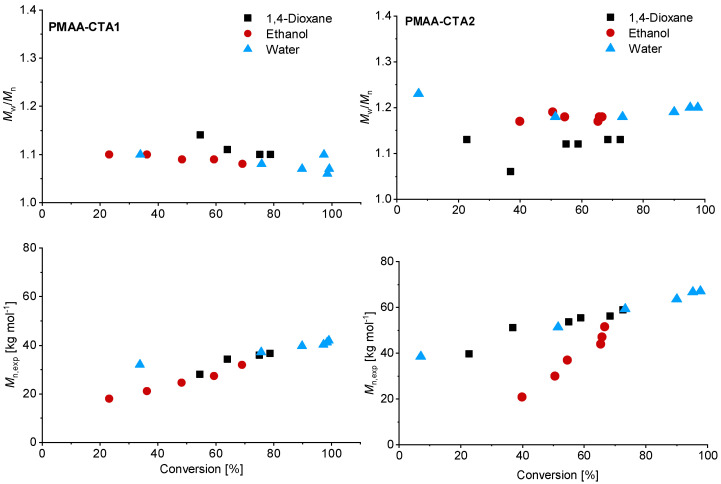
Reaction time versus conversion of the RAFT polymerization of PMAA with CTA1 (**left**) and CTA2 (**right**) with a [MAA]/[CTA] = 120 and [CTA]/[ACVA] = 10. The SEC results were measured in an aqueous set up with PEO standards.

Although higher experimental *M*_n,exp_ were verified compared to the theoretical values, a linear trend of *M*_n_ with conversion was achieved (Figure 3). The higher *M*_n, exp_ values are not surprising since the values were measured via SEC against PEO standards. Relatively low dispersities were confirmed in all solvents, although using water led to the lowest dispersity for the PMAA polymerization with CTA1 (*M*_w_/*M*_n_ = 1.07).

Similar observations were made for the polymerization with CTA2. The inhibition period changed for water to 1 h while the polymerization in ethanol confirmed measurable monomer consumption at this time. These observations are not surprising, since the CTA2 is more hydrophobic and therefore less soluble in water. This results in a lower reaction rate and longer inhibition phase, since fragmentations proceed more slowly. However, after 6 h almost full conversion in water was confirmed. The polymerization in ethanol led to just 67% conversion and in 1,4-dioxane to 73% conversion. SEC measurements showed narrow molar mass distributions in all cases and can be found in Figure 3. An overview about the PMAA polymerization of CTA1 and CTA2 can be found in the Supplementary Information (Table S1). Since all polymerizations showed narrow dispersities, it can be concluded that the solvent has a strong impact on the kinetics, however, the dispersity is less affected.

So far, the PMAA polymerization in water showed the fastest reaction rates and the narrowest molar mass distribution. Kuchta et al. investigated the effect of the propagation rate coefficient *k*_p_ in dependency of the solvent [36,37]. MAA tends to self-associate in bulk forming cyclic dimers. In organic media, however, this self-association step has to compete with the monomer-solvent complexes. This is caused by the hydrogen bonds between the carboxylic groups of monomers and the polar solvent. This self-association in organic media is more pronounced than in water, leading to a higher activation energy and therefore to reduced reaction rates in organic media compared to water [36,37].

**Figure 4:**
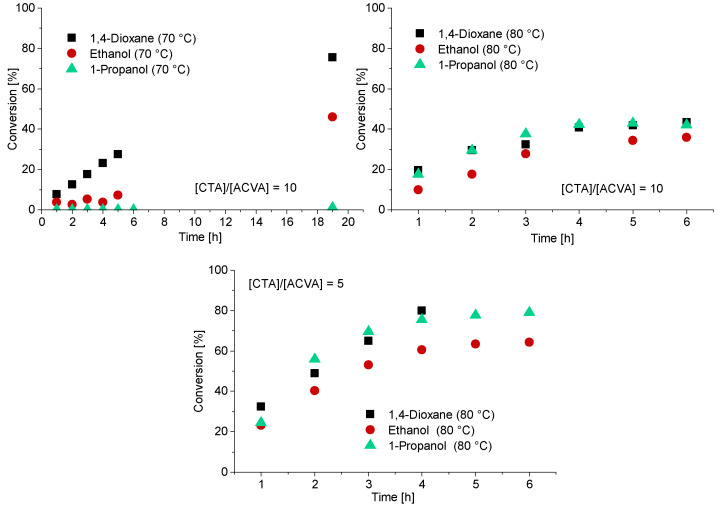
Reaction time versus conversion of the RAFT polymerization of PMAA with CTA3 and a targeted DP of [MAA]/[CTA3] = 120 and a molar ratio of [CTA3]/[ACVA] = 10. For the polymerizations at 80 °C a kinetical study with a molar ratio of [CTA3]/[ACVA] = 10 and 5 is displayed.

In case of the CTA3, water is a non-solvent. Therefore, polymerization with CTA3 was conducted in ethanol, 1-propanol and 1,4-dioxane at 70 °C and 80 °C. For the experiments at 80 °C a [CTA]/[ACVA] ratio of 5 and 10 was investigated. A targeted DP of 120 was adjusted for all experiments (Figure 4). The polymerizations at 70 °C led to low conversions and only in the case of 1,4-dioxane to a conversion of 75% after 19.5 h. To increase the reaction rate and thus the conversion, the reaction temperature was increased to 80 °C. The reaction rates of the polymerizations in ethanol and 1-propanol indeed increased and resulted in the inhibition phase being shortened and conversion being generated after only one hour. However, the conversion was below 50% in all cases. Increasing the initiator concentration to double the initial amount leads to fast reaction rates and high conversions after 4 h. The polymerizations in 1-propanol and 1,4-dioxane show the best reaction conditions, most probably due to better solubilization of the CTA. It should be noted that in case of the polymerization in 1,4-dioxane there were considerable viscosity problems, and it was therefore no longer possible to take samples after 4 h. The polymerization of PMAA with CTA3 in 1-propanol was the most promising reaction with conversions of approximately 80% within 4 h.

**Figure 5:**
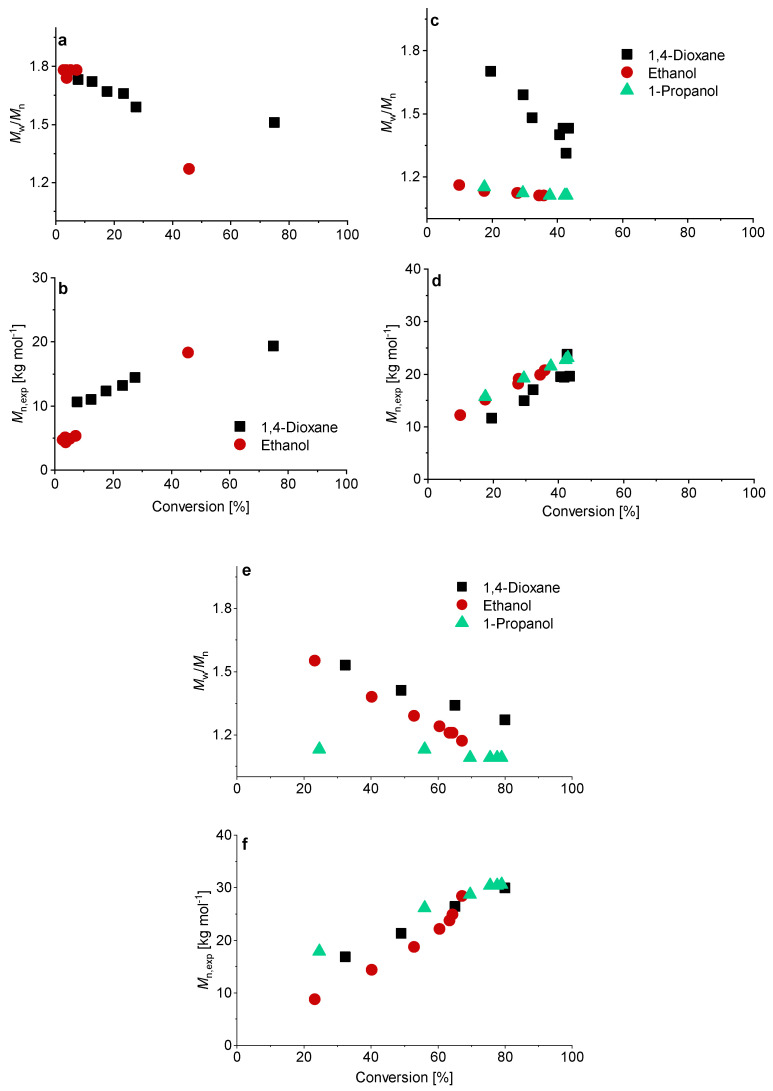
Conversion versus experimental number-average molar mass *M*_n, exp_ and *M*_w_/*M*_n_ of the RAFT polymerization of PMAA with CTA3 and a targeted DP of [MAA]/[CTA] = 120 and a molar ratio of [CTA]/[ACVA] = 10 at 70 °C (**a**,**b**). For the polymerizations at 80 °C a kinetical study with a molar ratio of [CTA]/[ACVA] = 10 (**c**,**d**) and 5 (**e**,**f**) is presented.

SEC measurements showed a linear relationship between conversion and molecular weight in all cases (Figure 5). The polymerization of PMAA with CTA3 in 1-propanol with a [CTA]/[ACVA] molar ratio = 5 at 80 °C showed so far the best reaction conditions and control (molar mass distribution (*M*_w_/*M*_n_ = 1.09)). A detailed overview can be found in Table S2.

Briefly, the polymerizations of MAA with CTA1 and CTA2 show the best reaction conditions in water. Both polymerizations have the advantage that they can be carried out in a green solvent. However, as a stabilizer block for the styrene polymerization, these blocks can reach their limit above a certain concentration of styrene, since these stabilizer blocks are not hydrophobic enough to stabilize the latex above a certain concentration. Therefore, the synthesis of the PMAA stabilizing block with CTA3 is a good alternative for the styrene polymerization. In the following, the influence of the three different stabilizer blocks in the emulsion polymerization of styrene will be investigated in detail.

### 3.2. Kinetics of the Synthesis of PMAA-b-PS with CTA1 via RAFT Emulsion Polymerization

The synthesis of the second block of PMAA-*b*-PS was conducted via RAFT emulsion polymerization in water. The PMAA homopolymer was used as both controlling and stabilizing agent. It is worth mentioning that the polymerization of the second block with CTA1 and CTA2 can be conducted in a one-pot synthesis, since the RAFT solution and emulsion polymerization can both be performed in water and since polymerization in the first step reaches full conversion. Several investigations on the one-pot synthesis with CTA1 have already been published [21,22,23,38]. Within a few hours, a diblock copolymer in water can be synthesized without intermediate precipitation steps of the homopolymer.

One of the advantages of the emulsion polymerization is the good heat transfer and low viscosity even at higher molecular weights, therefore, this technique was chosen to produce polymers with high molecular weights with the lowest possible by-products and low energy consumption.

**Figure 6:**
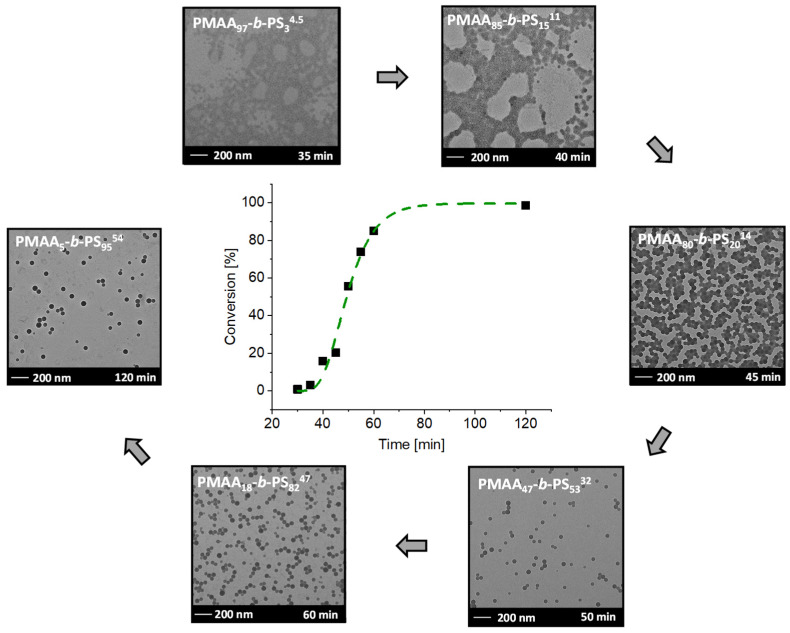
Conversion versus reaction time of the RAFT emulsion polymerization of PMAA_5_-*b*-PS_95_^54^ with CTA1 and a targeted DP of [MAA]/[CTA] = 30 and [Sty]/[CTA] = 502 and a molar ratio of [CTA]/[ACVA] = 5 at 80 °C and the corresponding TEM images after a specific time. The subscripts in the designation of the polymers represent the molar fraction of the respective block in wt% and the superscript represents the overall molecular weight in kDa.

The main work focused on the results of Chaduc et al. [21] conducting the reactions at a pH value of 2.5 and a [CTA]/[ACVA] molar ratio of 5. In a typical emulsion experiment, the macro-RAFT agent, ACVA and NaHCO_3_ (increases the water solubility of ACVA) were dissolved in water. Afterwards, styrene was added to the reaction, which was proceeded at a pH of 2.5 and a [CTA]/[ACVA] molar ratio of 5. The first experiments were conducted with CTA1 and a solid concentration of 20 wt%. The molar weight fraction of styrene was targeted to be 95% (PMAA_5_-*b*-PS_95_^54^). Samples were taken after 30, 35, 40, 45, 50, 55, 60, and 120 min. An inhibition period of around 30 min can clearly be seen (see Figure 6).

The inhibition period is described as the time, where no monomer conversion is obtained. More precisely, it is the time of the radical transfer to the MAA groups of the RAFT agent or the exit of the (macromolecular) R groups from the RAFT agent [39,40]. Before micellar nucleation the chain growth is slow as it can be seen during the time between 30–45 min. After micellar nucleation the polymerization rate increases drastically as demonstrated from the samples withdrawn after 45 and 50 min. The conversion increased by 36% in just 5 min. Therefore, it can be concluded that the micellar nucleation is finished after 40–45 min approximately. The fast conversion is due to the mechanistic transition from macro-CTA polymerization in the aqueous phase to macro-CTA polymerization in the micelles after reaching a critical PS block length. Full conversion is achieved within 2 h reaction time.

With time, conversion, molecular weight *M*_n_ and the radius of the micelles increased (Table S3). After full conversion a molecular weight of *M*_n,theo_ = 54.4 kDa (calculated according to the determined conversion from NMR spectroscopy, see Equation (1) in the experimental section)), a micelle radius of 29 ± 3 nm and a dispersity *M*_w_/*M*_n_ of 1.33 was achieved, proving a good control of the emulsion polymerization. Even at a very low molar weight ratio of just 5 wt% of the PMAA stabilizer block, stable micelles were clearly obtainable. They, furthermore, did not coalesce as is visible in the TEM images (Figure 6). Similar morphologies were observed for PMAA_3_-*b*-PS_97_^107^ with the same macro-CTA with a targeted DP of [MAA]/[CTA] = 30 and [Sty]/[CTA] = 1002. The resulting *M*_n,theo_ was obtained with 102 kDa, a *M*_w_/*M*_n_ of 1.42 and a micelle radius of 41 ± 3 nm for diblock copolymer. Clearly, the size of the micelles increases with higher molecular weight. Even though other morphologies are thermodynamically more favorable according to the packing parameter [41], kinetically-trapped spheres result almost in all cases using polystyrene as a major block even though highly asymmetric diblock compositions are targeted [20,22,24,42,43]. The only exception was the synthesis of PMAA_40_-*b*-PS_60_^46^ at 20 wt% prepared in a one-pot reaction using CTA1. Besides spherical particles, also worm-like micelles were observed. The corresponding TEM image can be found in the Supplementary Information (Figure S9). In previous publications, other morphologies such as fibers and vesicles were observed using a PMAA-*co*-PEO macro RAFT agent [44,45]. The ionization degree of PMAA as well as the molar mass played a crucial role in the transition of these morphologies. E.g., fiber morphologies were observed at a weight ratio from hydrophilic to hydrophobic of 70/30 to 80/20 at a pH of 5 [44,45]. In our case, a PMAA_40_-*b*-PS_60_^46^ diblock copolymer was synthesized with a weight fraction of the hydrophilic block of 60% at a pH of 2.5. In here, we can assume that only a low number of PMAA units were ionized, therefore the block is less hydrophilic than in the case of pH = 5. However, the weight fraction of the hydrophilic block was higher than in the cases of the literature where other morphologies than spheres were observed. Obviously, the ratio of the hydrophilic block as well as the degree of ionization play an important role to obtain other morphologies [44,45,46]. The previous experiments showed that polymerizations at a solid concentration of 20 wt% were well controlled even at high DPs of styrene. In the next step, the solid content of the emulsion polymerization (and hence also of the resulting dispersion) is investigated. The question is thus: how much water can actually be saved within the emulsion while still ensuring a well-controlled polymerization? Consequently, the PMAA chain extension at high solid concentrations is analyzed for each CTA system.

**Figure 7:**
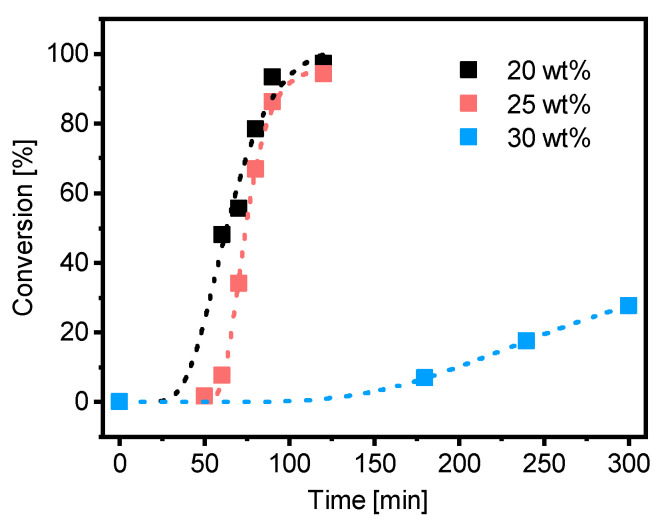
Conversion versus time of the RAFT emulsion polymerization of PMAA_10_-*b*-PS_90_^45^ with CTA1 and a targeted DP of [Sty]/[CTA] = 400 and a molar ratio of [CTA]/[ACVA] = 5 at 80 °C and a solid concentration of 20, 25 and 30 wt%.

The PMAA_10_-*b*-PS_90_^45^ polymerization with PMAA-CTA1 (DP = 50, *M*_w_/*M*_n_ = 1.25) was conducted at 20, 25 and 30 wt% in water at 80 °C. An overview of all emulsion polymerizations can be found in the Supplementary Information, Table S11. For all experiments a targeted styrene DP of 400 and a targeted *M*_n,theo_ of 45 kDa was set. A kinetic study of the synthesis showed that the polymerization in 20 and 25 wt% reached full conversion within 2 h, however the polymerization at 30 wt% reached less than 30% conversion after 5 h (Figure 7). Spherical morphologies were observed in all experiments. SEC data as well as information about the morphology and the size of the particles can be found in Tables S4–S6 and the TEM images in Figures S1–S3.

**Figure 8:**
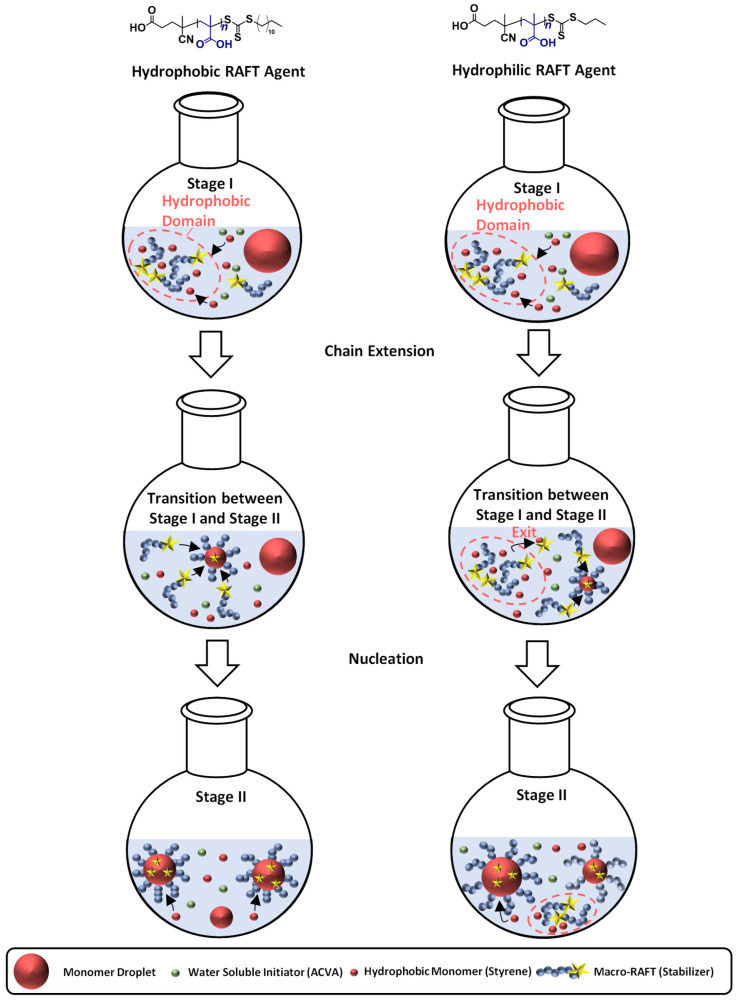
Illustration of the particle formation during an emulsion polymerization for a hydrophilic and hydrophobic RAFT agent as stabilizer with the three characteristics steps. Stage I shows the formation of micellar particles, Stage II the polymerization within the micelles and the transition state between both stages. The blue chains represent the stabilizing chain, the red chain the hydrophobic chain, the green circles label the initiator, the yellow star represents the active RAFT group, and the big red circles represent monomer swollen particles.

Polymerization of PMAA-*b*-PS with PMAA-CTA1 as a stabilizer at a solid concentration of 30 wt% shows a very slow polymerization rate. The RAFT stabilizing agent with a propyl group is probably still too hydrophilic to polymerize such a large concentration of styrene. An illustration of the RAFT emulsion polymerization with a hydrophobic and a hydrophilic RAFT agent can be seen in Figure 8. Generally, the emulsion polymerization is divided into three stages: the formation of micellar particles, the polymerization within the micelles and the consumption of the residual monomer in the micelles when the bigger droplets have vanished (Stage III is not shown in Figure 8). In a typical RAFT emulsion polymerization, the emulsion consists of a water-immiscible monomer, a water-soluble initiator, a macro-CTA as a stabilizer (replacing the surfactant in a conventional emulsion polymerization) and water. The monomer droplets have a size of approximately 10 µm and a number density of around 10^12^–10^14^ dm^−3^ in the aqueous medium. In case the concentration of the stabilizer block is higher than the critical micelle concentration (CMC), which is mostly the case for micellar nucleation, also micelles are present in the system with a size of approximately 5–10 nm and a number density of around 10^19^–10^21^ dm^−3^.

In case for a hydrophobic RAFT agent, the water-soluble initiator (in here ACVA) starts the polymerization and reacts with monomers (here styrene) in the continuous phase to form oligomer radicals which can either enter preexisting micelles or form new ones. The polymerization was conducted under acidic conditions, in order to form hyper-coiled structures of PMAA that intrinsically contain hydrophobic domains and thus enhance the local styrene concentration in the vicinity of the PMAA macro-RAFT [21,47]. The particle number increases in the first stage as the micelles convert into particles and the rate of polymerization increases due to an increase in the number of polymerization loci. By the end of Stage I all free macro-CTA chains are located in micelles and particle nucleation stops. Stage II sets in when only monomer droplets and polymer particles are present in the system. The polymerization continues inside the particles with monomer migration from the monomer droplets through the aqueous phase into polymeric particles to refill the polymerized monomer. As a result, a steady state between the monomer migration rate and the polymerization rate is accomplished, making the monomer concentration in the polymer micelles constant. The active radical within the particle keeps growing until it terminates upon entry of another radical or until it exits the particle into the aqueous phase. As this process is especially unlikely in the later periods of Stage II, the particles increase in size due to further absorption of monomer and an increase of chain length. At the end of Stage II, all monomer droplets disappear and the leftover monomer in the loci of polymerization is consumed (Stage III). The concentration of the remaining monomer in the polymer particles hence decreases with an increase in conversion, viscosity, and polymer volume fraction. At high conversions, propagation and termination become diffusion controlled, resulting in a speed up of the polymerization rate. Moreover, exit of the small radical becomes less likely, thereby allowing the particles to have more than one active radical without terminating instantly. Eventually, the propagation rate slows down (due to monomer depletion) resulting in a decrease of the rate of polymerization. The polymerization ends as almost all of the monomer is depleted [40,48,49,50,51,52].

**Figure 9:**
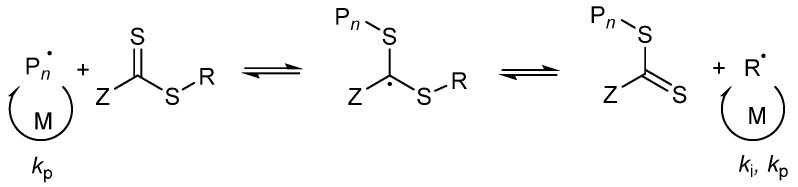
Illustration of the pre-equilibrium of the RAFT mechanism.

In case of a hydrophilic RAFT agent, for example the CTA1 at very high solid concentrations of styrene (above 30 wt%), slow nucleation and polymerization occurs. The group of Zetterlund et al. described that this phenomenon is caused by the Z-group induced RAFT exit [53]. The Z-group RAFT species is produced by the addition of a radical, entering a precursor particle and fragmentation afterwards to create a PMAA radical (pre-equilibrium of the RAFT mechanism, see Figure 9). The hydrophilic RAFT species, in case only a few units of styrene were added, might exit the hydrophobic domains (shown in Figure 8) due to their different polarities. The loss of RAFT agent from the polymerization loci reduces the chain extension rate, thus, less amphiphilic chains will be created, which impact the colloidal stability negatively, leading to larger and fewer particles [53].

In previous publications, some authors have described the long inhibition period as well as no polymerization in a RAFT emulsion polymerization due to the exit of the radical R˙ from the particle (R group cleavage of the RAFT agent in the pre-equilibrium step of the RAFT mechanism), where it can terminate in the aqueous phase with another initiator-derived radical or after reentry with a growing chain in a particle [40,54,55,56,57]. If this is the case, some particles will lose the propagating radical and need to be reinitiated again. This effect results in a lower number of particles [57]. The main difference between the R- and Z-group exit is that the R-group exit does not contain the RAFT functionality. The functionality remains in the particles.

The successfully controlled emulsion polymerization with more hydrophobic RAFT agents for the system of PAA-*b*-PS/PMAA-*b*-PS or similar polymer systems from previous publications (mentioned above) gave us incentive to use more hydrophobic RAFT agents as well and thus achieve polymerizations at higher weight percentages of the hydrophobic second monomer. To the best of our knowledge, no PMAA-*b*-PS polymers have yet been carried out at high solid concentrations above 30 wt%. In the following, the propyl group as the Z-group is exchanged with a butyl and dodecyl terminus.

### 3.3. The Synthesis of PMAA-b-PS with CTA2 via RAFT Emulsion Polymerization

The polymerization of PMAA_20_-*b*-PS_80_^46^ with PMAA macro-CTA2 was conducted at 35 and 40 wt% in water. Since the PMAA-CTA2 as well as the PMAA-*b*-PS polymer can be synthesized in water, a one-pot polymerization can be conducted. A DP of [Sty]/[CTA] = 360 was targeted. Compared to the PMAA_10_-*b*-PS_90_^45^ diblock copolymer with CTA1, the polymerization reached full conversion even at higher solid concentrations than 30 wt%. After 4 h, full conversion was achieved in all experiments. However, the polymerization at 40 wt% with CTA2 was very viscous after full conversion (see Figure S11). The SEC, DLS and TEM data can be found in Table 1. Very large particle radii of 85 nm and 96 nm were synthesized for 35 wt% and 40 wt% as measured by DLS, when replacing the propyl Z-group with a butyl Z-group. These results fit to the proposed mechanism from Zetterlund et al., which was also shown in Figure 8 for hydrophilic RAFT agents [53]. However, the very large deviation of *M*_n,exp_ from *M*_n,theo_ infers a not well-controlled polymerization. Therefore, the Z-group was further modified with a dodecyl terminus.

### 3.4. The Synthesis of PMAA-b-PS with CTA3 via RAFT Emulsion Polymerization

**Figure 10:**
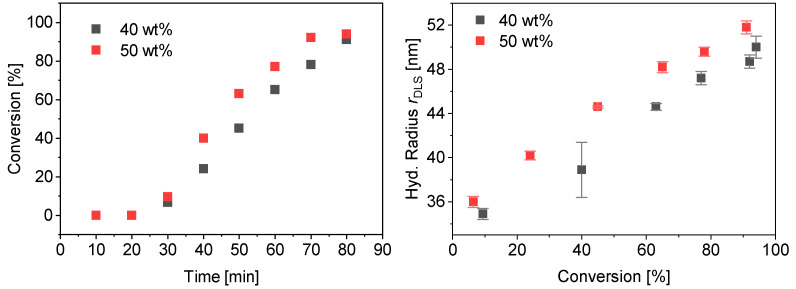
Conversion vs. time data for PMAA_20_-*b*-PS_80_^18^ with CTA3 at 40 wt% and 50 wt% (**left**) and average hydrodynamic radius measured by DLS against the conversion (**right**).

The polymerization of PMAA-*b*-PS with a CTA using a dodecyl terminus as a Z-group was tested. This CTA is more hydrophobic than CTA1 and CTA2. The R-group was not modified in any experiment. At first a diblock copolymer with a lower molecular weight was targeted. The synthesis of PMAA_20_-*b*-PS_80_^18^ with CTA3 (DP = 43, *M*_w_/*M*_n_ = 1.11, *M*_n,theo_ = 3.8 kDa) at 80 °C in water and a targeted styrene DP of 200 with a CTA3/ACVA molar ratio of 5 was carried out at 40 and 50 wt% solid concentration. Almost full conversion was achieved for both reactions already within 80 min (Figure 10). The hydrodynamic radius *r*_DLS_ increases linearly with conversion. The final dispersity *M*_w_/*M*_n_ = 1.23 for both polymerizations indicate a good control over the polymerization. More detailed information can be found in the Supplementary Information, Table S7 and Figures S4 and S5.

**Figure 11:**
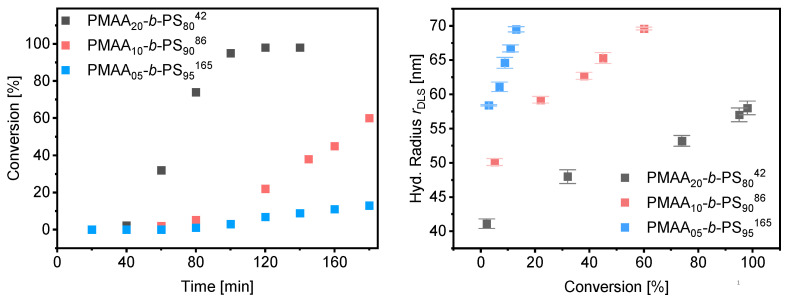
Conversion vs. time data for PMAA-*b*-PS with CTA3 at 50% (**left**) and average hydrodynamic radius measured by DLS against the conversion (**right**).

Since the polymerizations at 40 wt% as well as at 50 wt% worked in a controlled and fast manner, polymerizations were carried out at 50 wt% increasing the weight fraction of styrene as well as the total molecular weight. Three different block copolymer compositions were targeted with a weight fraction of 80%, 90% and 95% of styrene using the same macro-CTA3 in all experiments (DP = 95, *M*_w_/*M*_n_ = 1.09, *M*_n,theo_ = 8.5 kDa). The kinetic data of the polymerization rate and DLS measurements can be found in Figure 11. More detailed information about the morphology, SEM and TEM results can be found in Tables S8–S10 and Figures S6–S8. A well-controlled polymerization for the reaction with 50 wt% solid content and 80 wt% fraction of styrene was observed. Complete conversion was obtained after 2 h and a dispersity of *M*_w_/*M*_n_ = 1.22 was achieved. RI und UV detection of the SEC measurement overlapped proving that majority of the chains containing the RAFT group indicating successful chain extension (see Figure S10). The radius of the spherical particles increases linearly with conversion (Figure 10). As described for the mechanism before, the effect of the Z-group plays a significant role in the rate of emulsion polymerization. Both our results and those of Zetterlund et al. confirm the mechanism assumed above. Polymerizations with 90 and 95 wt% styrene resulted in conversions of 60% and 13% after 3 h, respectively. The polymerization of emulsions at 50 wt% requires a good stability of the colloids which, even with commercial surfactants, must ensure an extremely good control of the reaction and reach their limit. In this work it was shown that polymerization by RAFT with hydrophobic RAFT agents allows such a polymerization in a controlled manner.

## 4. Conclusions

The synthesis of PMAA-*b*-PS diblock copolymers at high solid concentrations was investigated by varying the Z-group of the RAFT agent from a *n*-propyl-, to *n*-butyl- and *n*-dodecyl- terminus, making the RAFT agent more hydrophobic. RAFT polymerization of MAA was carried out in water with a trithiocarbonate RAFT agent. A carboxyl group was chosen as the R-group for all experiments. Promising conditions for PMAA polymerizations were found for the propyl and butyl moieties in water (or 1-propanol in case of CTA3). For successive chain extension with PS, the polymerizations performed with a propyl or butyl Z-group could be carried out in a one-pot synthesis. However, these polymerizations resulted in very long polymerization times or uncontrolled conditions when styrene weight concentrations above 30 wt% were chosen. The replacement of the Z group with a dodecyl radical made it possible to carry out the polymerization of PMAA-*b*-PS under very well controlled conditions up to remarkably high solid contents of 50 wt% provided that the PMAA block is long enough. We could also confirm that the influence of the Z-group has a significant effect on the polymerization rate in the RAFT emulsion polymerization. With this synthesis method, we have found a reaction pathway that allows the use of green solvents and due to the high solid concentrations, this system is associated with less by-product and energy consumption. The chosen hydrophilic PMAA block not only offers the possibility of further post-modifications resulting in a variety of functionalized nanoparticles, but these block copolymers are also potential candidates for membrane technology.

## Figures and Tables

**Table 1:** Polymerization of PMAA_20_-*b*-PS_80_^46^ with CTA2 at 80 °C in water and a targeted styrene DP of 360 with a [CTA2]/[ACVA] molar ratio of 5. All reactions were polymerized for 4 h. *r*_TEM_ represents the number-average radius obtained by TEM and *r*_DLS_ by DLS. *M*_n,theo_ represents the theoretical number-average molecular weight of PMAA-*b*-PS. The dispersity *M*_w_/*M*_n_ was determined my SEC THF + 50 mmol TFA using universal PS calibration. The PMAA-CTA2 macro-CTA was determined with NMR and aqueous SEC using PEO calibration with *M*_n,theo_ = 8.7 kDa and a *M*_w_/*M*_n_ = 1.17.

(*w*/*w*) Solids [%]	Conversion [%]	*r*_TEM_ [nm]	*r*_DLS_ [nm]	*PDI*	*M*_n,theo_ [kDa]	*M*_n,exp_ [kDa]	*M*_w_ */M*_n_
35	99	61 ± 4	85.3 ± 0.5	0.09 ± 0.03	46	166	1.57
40	99	70 ± 5	96 ± 2	0.3 ± 0.2	46	158	1.61

## Data Availability

The data presented in this study are available on request from the corresponding author.

## Supplementary Materials

The following are available online at https://www.mdpi.com/article/10.3390/polym13213675/s1, Table S1: kinetic data of the PMAA polymerization with CTA1 and CTA2; Table S2: kinetic data of the PMAA polymerization with CTA3; Table S3: kinetic data of PMAA_5_-*b*-PS_95_^54^ with CTA1 at 20 wt%, Table S4: kinetic data of PMAA_10_-*b*-PS_90_^45^ with CTA1 at 20 wt%; Table S5: kinetic data of PMAA_10_-*b*-PS_90_^45^ with CTA1 at 25 wt%, Table S6: kinetic data of PMAA_10_-*b*-PS_90_^45^ with CTA1 at 30 wt%, Table S7: kinetic data of PMAA_20_-*b*-PS_80_**^18^** with CTA3 at 40 and 50 wt%; Table S8: kinetic data of PMAA_20_-*b*-PS_80_^42^ with CTA3 at 50 wt%; Table S9: kinetic data of PMAA_10_-*b*-PS_90_^86^ with CTA3 at 50 wt%, Table S10: kinetic data of PMAA_5_-*b*-PS_95_^165^ with CTA3 at 50 wt%; Table S11: overview of all PMAA-*b*-PS diblock copolymers and the corresponding macro-CTAs; Figure S1: TEM image of PMAA_10_-*b*-PS_90_^45^ with CTA1 at 20 wt%; Figure S2: TEM image of PMAA_10_-*b*-PS_90_^45^ with CTA1 at 25 wt%; Figure S3: TEM image of PMAA_10_-*b*-PS_90_^45^ with CTA1 at 30 wt%; Figure S4: TEM image of PMAA_20_-*b*-PS_80_^26^ with CTA3 at 40 wt%; Figure S5: TEM image of the PMAA_20_-*b*-PS_80_^26^ with CTA3 at 50 wt%; Figure S6: TEM image of PMAA_20_-*b*-PS_80_^42^ with CTA3; Figure S7: TEM image of PMAA_10_-*b*-PS_90_^86^ with CTA3; Figure S8: TEM image of PMAA_5_-*b*-PS_95_^165^ with CTA3; Figure S9: TEM image of PMAA_40_-*b*-PS_60_^46^ showing worm-like morphologies; Figure S10: SEC results of PMAA_20_-*b*-PS_80_^42^ witch CTA3 at 50 wt%; Figure S11: pictures of the polymerization of PMAA-*b*-PS with CTA2 and CTA3 at 40 wt%.
