# Supplementary material for: Synthesis of Poly(methacrylic acid)-block-Polystyrene Diblock Copolymers at High Solid Contents via RAFT Emulsion Polymerization
Source: Polymers (Basel). 2021 Oct 25;13(21):3675. doi: 10.3390/polym13213675 (PMC8588034; doi:10.3390/polym13213675)
Supplement: Supplementary file 1 [file polymers-13-03675-s001.zip › polymers-1428139-supplementary.pdf]

polymers

MDPI

Article

Synthesis of Poly(methacrylic acid)-*block*-Polystyrene Diblock Copolymers at High Solid Contents via RAFT Emulsion Polymerization

Iklima Oral [1], Larissa Grossmann [1], Elena Fedorenko [1], Jana Struck [1] and Volker Abetz [1,2,*]

[1] Institute of Physical Chemistry, Universität Hamburg, Grindelallee 117, 20146 Hamburg, Germany; iklima.oral@chemie.uni-hamburg.de (I.O.); larissa.grossmann@studium.uni-hamburg.de (L.G.); elena.fedorenko@chemie.uni-hamburg.de (E.F.); jana.struck@chemie.uni-hamburg.de (J.S.)
[2] Helmholtz-Zentrum Hereon, Institute of Membrane Research, Max-Planck-Straße 1, 21502 Geesthacht, Germany
* Correspondence: volker.abetz@hereon.de; Tel.: +49-40-42838-3460

Supplementary Materials

Citation: Oral, I.; Grossmann, L.; Fedorenko, E.; Struck, J.; Abetz, V. Synthesis of Poly(methacrylic acid)-*block*-Polystyrene Diblock Copolymers at High Solid Contents via RAFT Emulsion Polymerization. *Polymers* **2021**, *13*, 3675. https://doi.org/10.3390/polym13213675

Academic Editor: Leonard Ionut Atanase

Received: 3 October 2021
Accepted: 15 October 2021
Published: 25 October 2021

Publisher's Note: MDPI stays neutral with regard to jurisdictional claims in published maps and institutional affiliations.

Copyright: © 2021 by the authors. Submitted for possible open access publication under the terms and conditions of the Creative Commons Attribution (CC BY) license (https://creativecommons.org/licenses/by/4.0/).

Table S1. Kinetic Data of the Polymerization of PMAA at 70 °C and a molar ratio of [CTA]/[ACVA] = 10 and [MAA]/[CTA] = 120. $M_{n,theo}$ represents the theoretical number-average molecular weight of PMAA and $M_{n,exp}$ the experimental theoretical number-average molecular weight measured by SEC. The dispersity M_w/M_n was determined my aqueous SEC using PEO calibration. CTA1 represents the RAFT CTA with a propyl terminus as a Z-group and CTA2 the butyl terminus.

CTA	Solvent	Time [min]	Conversion [%]	$M_{n,theo}$ [kDa]	$M_{n,exp}$ [kDa]	M_w/M_n
CTA1	water	0	0	0	-	-
CTA1	water	60	33.9	3.59	32.1	1.10
CTA1	water	120	75.8	8.04	37.2	1.08
CTA1	water	180	89.8	9.52	39.7	1.07
CTA1	water	240	97.3	10.3	40.2	1.12
CTA1	water	300	98.5	10.4	41.2	1.06
CTA1	water	360	99.1	10.5	42.0	1.07
CTA1	ethanol	0	0	0	-	-
CTA1	ethanol	60	0	0	-	-
CTA1	ethanol	120	23.2	2.46	17.9	1.10
CTA1	ethanol	180	36.3	4.46	21.2	1.10
CTA1	ethanol	240	48.3	5.93	24.6	1.09
CTA1	ethanol	300	59.4	7.30	27.2	1.09
CTA1	ethanol	360	69.1	8.50	31.9	1.08
CTA1	1,4-dioxane	0	0	0	-	-
CTA1	1,4-dioxane	60	30.5	3.74	-	-
CTA1	1,4-dioxane	120	54.6	6.71	28.8	1.14
CTA1	1,4-dioxane	180	64.0	7.87	34.2	1.11
CTA1	1,4-dioxane	240	75.2	9.24	35.9	1.10
CTA1	1,4-dioxane	300	78.8	9.69	36.8	1.10
CTA2	water	0	0	0	-	-
CTA2	water	60	7.00	0.743	-	-
CTA2	water	120	51.5	5.47	51.3	1.18
CTA2	water	180	73.3	7.78	59.2	1.18
CTA2	water	240	90.1	9.56	63.4	1.19
CTA2	water	300	95.2	10.1	66.6	1.20
CTA2	water	360	97.8	10.4	66.9	1.20
CTA2	ethanol	0	0	0	-	-
CTA2	ethanol	60	40.0	4.24	20.7	1.17
CTA2	ethanol	120	50.6	5.37	30.0	1.19
CTA2	ethanol	180	54.7	5.81	36.8	1.19
CTA2	ethanol	240	65.4	6.95	43.9	1.17
CTA2	ethanol	300	65.9	7.00	46.9	1.18
CTA2	ethanol	360	66.7	7.08	51.4	1.18
CTA2	1,4-dioxane	0	0	0	-	-
CTA2	1,4-dioxane	60	22.8	2.42	39.4	1.13
CTA2	1,4-dioxane	120	36.9	3.92	50.8	1.06
CTA2	1,4-dioxane	180	55.1	5.85	53.2	1.12
CTA2	1,4-dioxane	240	58.9	6.26	54.9	1.12
CTA2	1,4-dioxane	300	68.5	7.27	55.8	1.13
CTA2	1,4-dioxane	360	72.6	7.71	58.5	1.13

Table S2. Kinetic Data of the Polymerization of PMAA with CTA3 with a molar ratio of [MAA]/[CTA3] = 120. $M_{n,theo}$ represents the theoretical number-average molecular weight of PMAA and $M_{n,exp}$ the experimental theoretical number-average molecular weight measured by SEC. The dispersity M_w/M_n was determined my aqueous SEC using PEO calibration. CTA3 represents the RAFT CTA with a dodecyl- terminus as a Z-group.

[CTA]/ [ACVA]	Solvent	Time [min]	Temperature [°C]	Conversion [%]	$M_{n,theo}$ [kDa]	$M_{n,exp}$ [kDa]	M_w/M_n
10	1,4-dioxane	0	70	0	0	-	-
10	1,4-dioxane	60	70	8.00	0.968	10.6	1.73
10	1,4-dioxane	120	70	12.5	1.35	11.0	1.72
10	1,4-dioxane	180	70	17.6	1.87	12.3	1.67
10	1,4-dioxane	240	70	23.2	2.49	13.2	1.66
10	1,4-dioxane	300	70	27.5	2.95	14.4	1.59
10	1,4-dioxane	1170	70	75.1	8.06	19.3	1.51
10	ethanol	0	70	0	0	-	-
10	ethanol	60	70	3.80	0.410	4.30	1.74
10	ethanol	120	70	2.70	0.295	4.70	1.78
10	ethanol	180	70	5.20	0.557	4.80	1.78
10	ethanol	240	70	3.70	0.393	5.10	1.78
10	ethanol	300	70	7.20	0.770	5.30	1.78
10	ethanol	1170	70	45.8	4.91	18.3	1.27
10	1-propanol	0	70	0	0	-	-
10	1-propanol	60	70	0	0	-	-
10	1-propanol	120	70	0	0	-	-
10	1-propanol	180	70	0	0	-	-
10	1-propanol	240	70	0	0	-	-
10	1-propanol	300	70	0	0	-	-
10	1,4-dioxane	0	80	0	0	-	-
10	1,4-dioxane	60	80	19,5	2.10	11.6	1.70
10	1,4-dioxane	120	80	29.6	3.17	14.9	1.59
10	1,4-dioxane	180	80	32.3	3.47	17.0	1.48
10	1,4-dioxane	240	80	40.7	4.37	19.5	1.40
10	1,4-dioxane	300	80	41.8	4.49	19.4	1.43
10	1,4-dioxane	360	80	43.5	4.66	19.6	1.43
10	ethanol	0	80	0	0	-	-
10	ethanol	60	80	9.20	0.990	5.50	1.73
10	ethanol	120	80	11.4	1.23	6.90	1.64
10	ethanol	180	80	11.4	1.23	7.10	1.65
10	ethanol	240	80	9.86	1.06	7.30	1.65
10	ethanol	300	80	13.9	1.49	7.60	1.64
10	ethanol	360	80	12.5	1.34	7.70	1.63
10	1-propanol	0	80	0	0	-	-
10	1-propanol	60	80	17.6	1.89	15.7	1.15
10	1-propanol	120	80	29.5	3.16	19.2	1.12
10	1-propanol	180	80	37.7	4.05	21.5	1.11
10	1-propanol	240	80	42.3	4.54	22.7	1.11
10	1-propanol	300	80	42.9	4.61	23.1	1.11
10	1-propanol	360	80	42.0	4.51	23.1	1.16
5	1,4-dioxane	0	80	0	0	-	-
5	1,4-dioxane	60	80	32.4	3.48	16.8	1.53
5	1,4-dioxane	120	80	49.1	5.27	21.3	1.41

[CTA]/[ACVA]	Solvent	Time [min]	Temperature [°C]	Conversion [%]	$M_{n,theo}$ [kDa]	$M_{n,exp}$ [kDa]	M_w/M_n
5	1,4-dioxane	180	80	65.1	6.99	26.4	1.34
5	1,4-dioxane	240	80	80.1	8.59	29.9	1.27
5	ethanol	0	80	0	0	-	-
5	ethanol	60	80	23.2	2.49	8.70	1.55
5	ethanol	120	80	40.3	4.32	14.3	1.38
5	ethanol	180	80	53.0	5.69	18.7	1.29
5	ethanol	240	80	60.5	6.49	22.1	1.24
5	ethanol	300	80	63.5	6.81	23.8	1.21
5	ethanol	360	80	64.4	6.91	24.9	1.21
5	1-propanol	0	80	0	0	-	-
5	1-propanol	60	80	24.6	2.64	17.9	1.13
5	1-propanol	120	80	56.0	6.01	26.1	1.13
5	1-propanol	180	80	69.7	7.48	28.7	1.09
5	1-propanol	240	80	75.6	8.11	30.4	1.09
5	1-propanol	300	80	77.7	8.34	30.4	1.09
5	1-propanol	360	80	79.0	8.47	30.6	1.09

Table S3. Kinetic Data of the Polymerization of $PMAA_5$-*b*-PS_{95}[54] with CTA1 at 80 °C in water and a targeted styrene DP of 502 with a [CTA1]/[ACVA] molar ratio of 5. r_{TEM} represents the number-average radius obtained by TEM. $M_{n,theo}$ represents the theoretical number-average molecular weight of PMAA-*b*-PS and $M_{n,theo*}$ the theoretical number-average molecular weight of PMMA-*b*-PS. The dispersity M_w/M_n was determined my SEC THF using PS calibration.

Time [min]	Conversion [%]	r_{TEM} [nm]	$M_{n,theo}$ [kDa]	$M_{n,theo*}$ [kDa]	$M_{n,exp}$ [kDa]	M_w/M_n
35	3.1	-	4.48	4.90	-	-
40	16	15 ± 2	11.1	11.5	12.5	1.13
45	20	18 ± 2	13.5	14.0	25.5	1.26
50	56	25 ± 2	31.9	32.3	43.2	1.30
55	74	24 ± 3	42.0	42.0	56.2	1.30
60	85	27 ± 3	47.4	47.8	64.2	1.27
120	99	29 ± 3	54.4	54.8	69.1	1.33

Table S4. Kinetic Data of the Polymerization of $PMAA_{10}$-*b*-PS_{90}[45] with CTA1 at 80 °C in water and a targeted MAA DP of 50 and a styrene DP of 400 with a [CTA1]/[ACVA] molar ratio of 5 at 20 wt%. r_{TEM} represents the number-average radius obtained by TEM and r_{DLS} by DLS. $M_{n,theo}$ represents the theoretical number-average molecular weight of PMAA-*b*-PS and $M_{n,theo*}$ the theoretical number-average molecular weight of PMMA-*b*-PS. The dispersity M_w/M_n was determined my SEC THF using PS calibration after methylation.

Time [min]	Conversion [%]	r_{DLS} [nm]	*PDI*	r_{TEM} [nm]	$M_{n,theo}$ [kDa]	$M_{n,theo*}$ [kDa]	$M_{n,exp}$ [kDa]	M_w/M_n
60	48	24 ± 1	0.3 ± 0.2	-	22.3	22.7	-	-
70	56	28.88 ± 0.06	0.09 ± 0.02	-	25.8	26.2	30.0	1.17
80	78	31.79 ± 0.06	0.08 ± 0.02	-	36.3	36.3	53.8	1.28
90	93	31.9 ± 0.2	0.07 ± 0.02	-	43.2	43.2	66.7	1.28
120	97	32.0 ± 0.5	0.04 ± 0.02	24 ± 3	45.0	44.9	69.5	1.26

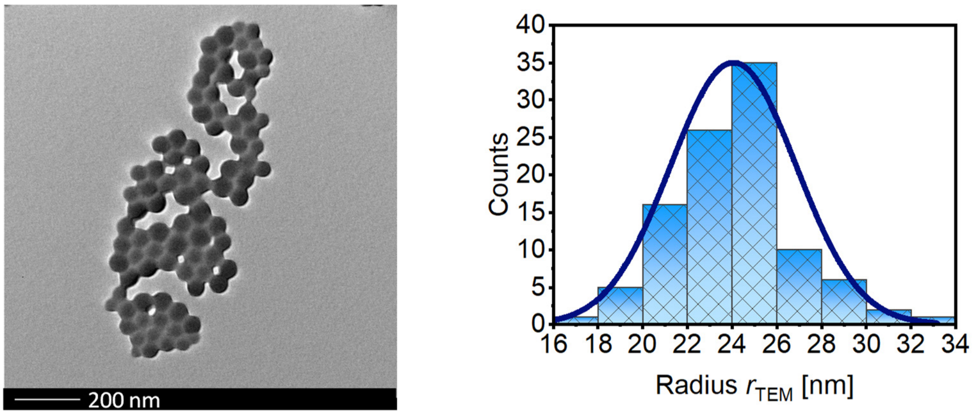

Figure S1. TEM image (left) and histogram (right) of the number-average radius of the PMAA$_{10}$-*b*-PS$_{90}^{45}$ with CTA1 copolymer and 20 wt% after 120 minutes reaction time.

Table S5. Kinetic Data of the Polymerization of PMAA$_{10}$-*b*-PS$_{90}^{45}$ with CTA1 at 80 °C in water and a targeted MAA DP of 50 and a styrene DP of 400 with a [CTA1]/[ACVA] molar ratio of 5 at 25 wt%. r_{TEM} represents the number-average radius obtained by TEM and r_{DLS} by DLS. $M_{n,theo}$ represents the theoretical number-average molecular weight of PMAA-*b*-PS and $M_{n,theo^*}$ the theoretical number-average molecular weight of PMMA-*b*-PS. The dispersity M_w/M_n was determined my SEC THF using PS calibration after methylation.

Time [min]	Conversion [%]	r_{DLS} [nm]	*PDI*	r_{TEM} [nm]	$M_{n,theo}$ [kDa]	$M_{n,theo^*}$ [kDa]	$M_{n,exp}$ [kDa]	M_w/M_n
50	1.8	-	-	-	1.09	1.10	-	-
60	7.5	26 ± 1	0.4 ± 0.2	-	3.84	3.89	-	-
70	34	28.88 ± 0.06	0.09 ± 0.02	-	15.9	16.2	27.3	1.35
80	67	30.6 ± 0.3	0.07 ± 0.02	24 ± 2	31.1	31.5	51.7	1.35
90	86	32.5 ± 0.6	0.08 ± 0.03	-	39.8	40.4	70.8	1.40
120	94	33.1 ± 0.3	0.04 ± 0.01	29 ± 3	43.6	44.2	71.0	1.50

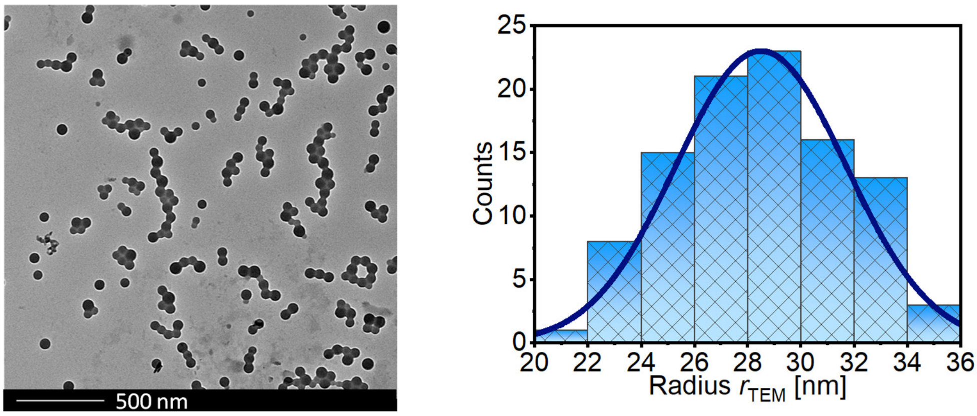

Figure S2. TEM image (left) and histogram (right) of the number-average radius of the PMAA$_{10}$-*b*-PS$_{90}^{45}$ with CTA1 copolymer and 25 wt% after 120 minutes reaction time.

Table S6. Kinetic Data of the Polymerization of $PMAA_{10}$-*b*-PS_{90}^{45} with CTA1 at 80 °C in water and a targeted MAA DP of 50 and a styrene DP of 400 with a [CTA1]/[ACVA] molar ratio of 5 at 30 wt%. r_{TEM} represents the number-average radius obtained by TEM and r_{DLS} by DLS. $M_{n,theo}$ represents the theoretical number-average molecular weight of PMAA-*b*-PS and $M_{n,theo^*}$ the theoretical number-average molecular weight of PMMA-*b*-PS. The dispersity M_w/M_n was determined my SEC THF using PS calibration after methylation.

Time [min]	Conversion [%]	r_{DLS} [nm]	*PDI*	r_{TEM} [nm]	$M_{n,theo}$ [kDa]	$M_{n,theo^*}$ [kDa]	$M_{n,exp}$ [kDa]	M_w/M_n
180	6.9	-	-	-	3.44	3.49	-	-
240	18	-	-	-	8.38	8.50	-	-
300	28	42.1 ± 0.3	0.56 ± 0.04	26 ± 2	13.0	13.2	26.0	1.38

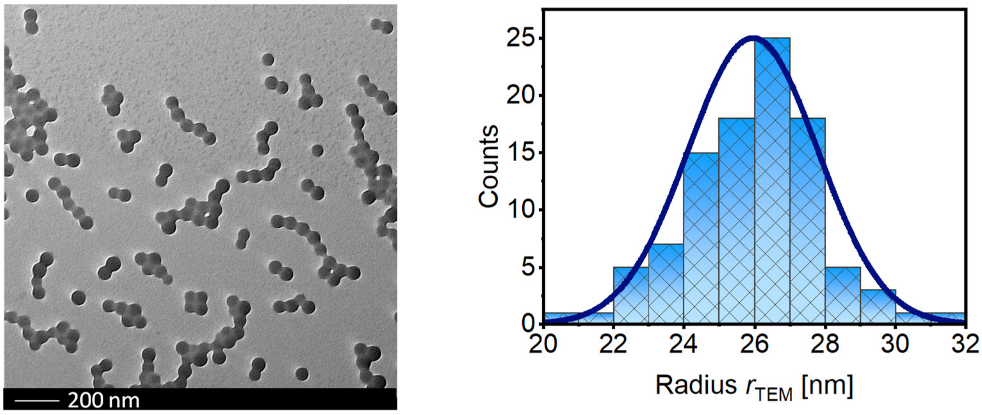

Figure S3. TEM image (left) and histogram (right) of the number-average radius of the $PMAA_{10}$-*b*-PS_{90}^{45} with CTA1 copolymer and 30 wt% after 300 minutes reaction time.

Table S7. Polymerization of $PMAA_{20}$-*b*-PS_{80}^{18} with CTA3 at 80 °C in water and a targeted styrene DP of 200 with a [CTA3]/[ACVA] molar ratio of 5. r_{TEM} represents the number-average radius obtained by TEM and r_{DLS} by DLS. $M_{n,theo}$ represents the theoretical number-average molecular weight of PMAA-*b*-PS. The dispersity M_w/M_n was determined my SEC THF + 35 mmol TFA using universal PS calibration. The PMAA-CTA3 macro-CTA was synthesized in ethanol and determined with NMR and aqueous SEC using PEO calibration with $M_{n,theo}$ = 3.8 kDa and a M_w/M_n = 1.11.

(*w*/*w*) solids [%]	Time [min]	Conversion [%]	r_{TEM} [nm]	r_{DLS} [nm]	*PDI*	$M_{n,theo}$ [kDa]	$M_{n,exp}$ [kDa]	M_w/M_n
40	10	0	-	-	-	3.8	-	-
40	20	0	-	-	-	3.8	-	-
40	30	6.5	-	36.0 ± 0.5	0.15 ± 0.06	4.8	-	-
40	40	24	-	40.2 ± 0.4	0.03 ± 0.02	7.4	-	-
40	50	45	-	44.6 ± 0.1	0.07 ± 0.01	10	35	1.17
40	60	65	-	48.2 ± 0.5	0.05 ± 0.05	13	46	1.20
40	70	78	-	49.6 ± 0.4	0.02 ± 0.02	15	53	1.20
40	80	91	45 ± 3	51.8 ± 0.6	0.05 ± 0.02	17	59	1.23
50	10	0	-	-	-	3.8	-	-
50	20	0	-	-	-	3.8	-	-
50	30	9.4	-	34.9 ± 0.5	0.14 ± 0.09	5.3	-	-
50	40	40	-	38.9 ± 2.5	0.21 ± 0.23	9.7	35	1.18
50	50	63	-	44.6 ± 0.3	0.06 ± 0.05	13	46	1.18
50	60	77	-	47.2 ± 0.6	0.03 ± 0.02	15	55	1.20
50	70	92	-	48.7 ± 0.6	0.04 ± 0.03	17	62	1.21
50	80	94	41± 2	50 ± 1	0.06 ± 0.05	18	63	1.23

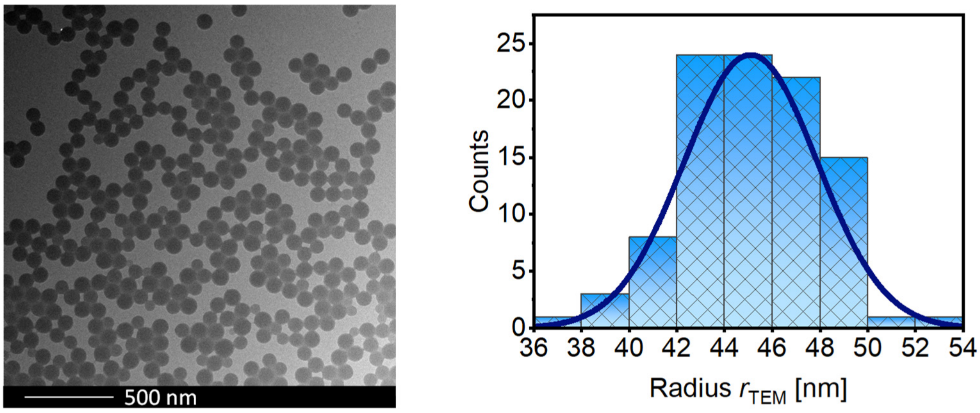

Figure S4. TEM image (left) and histogram (right) of the number-average radius of the $PMAA_{20}$-*b*-PS_{80}[18] with CTA3 copolymer and 40 wt%.

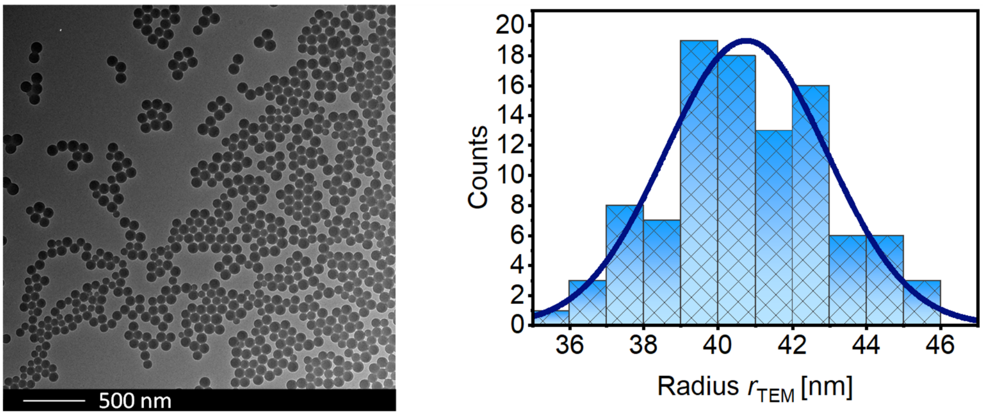

Figure S5. TEM image (left) and histogram (right) of the number-average radius of the $PMAA_{20}$-*b*-PS_{80}[18] with CTA3 copolymer and 50 wt%.

Table S8. Kinetic Data of the Polymerization of $PMAA_{20}$-*b*-PS_{80}[42] with CTA3 at 80 °C in water with a targeted styrene DP of 320 with a [CTA3]/[ACVA] molar ratio of 5 at 50 wt%. r_{TEM} represents the number-average radius obtained by TEM and r_{DLS} by DLS. $M_{n,theo}$ represents the theoretical number-average molecular weight of PMAA-*b*-PS and $M_{n,theo^*}$ the theoretical number-average molecular weight of PMMA-*b*-PS. The dispersity M_w/M_n was determined my SEC THF + 35 mmol TFA using universal PS calibration. The PMAA-CTA3 macro-CTA was synthesized in 1-propanol and determined with NMR and aqueous SEC using PEO calibration with $M_{n,theo}$ = 8.5 kDa and a M_w/M_n = 1.09.

**Time [min]**	**Conversion [%]**	r_{DLS} **[nm]**	*PDI*	r_{TEM} **[nm]**	$M_{n,theo}$ **[kDa]**	$M_{n,exp}$ **[kDa]**	M_w/M_n
20	0	-	-	-	-	-	-
40	2.2	41.1 ± 0.7	0.55 ± 0.05	-	9.2	-	-
60	32	48 ± 1	0.06 ± 0.04	32 ± 3	19	27.6	1.16
80	74	53.2 ± 0.8	0.05 ± 0.03	42 ± 4	33	50.3	1.20
100	95	57 ± 1	0.05 ± 0.05	42 ± 4	40	58.9	1.21
120	98	58 ± 1	0.02 ± 0.02	-	41	59.2	1.22
140	98	56.2 ± 0.8	0.05 ± 0.02	-	41	59.6	1.22

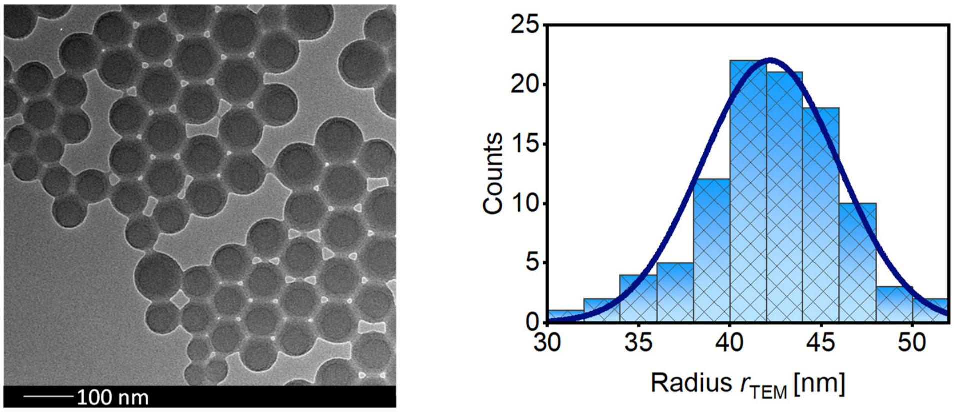

Figure S6. TEM image (left) and histogram (right) of the number-average radius of the of $PMAA_{20}$-*b*-PS_{80}^{42} after 100 minutes reaction time with CTA3 copolymer and 50 wt% and 80 % weight fraction of styrene.

Table S9. Kinetic Data of the Polymerization of $PMAA_{10}$-*b*-PS_{90}^{86} with CTA3 at 80 °C in water with a targeted styrene DP of 740 with a [CTA3]/[ACVA] molar ratio of 5 at 50 wt%. r_{TEM} represents the number-average radius obtained by TEM and r_{DLS} by DLS. $M_{n,theo}$ represents the theoretical number-average molecular weight of PMAA-*b*-PS and $M_{n,theo*}$ the theoretical number-average molecular weight of PMMA-*b*-PS. The dispersity M_w/M_n was determined by SEC THF + 35 mmol TFA using universal PS calibration. The PMAA-CTA3 macro-CTA was synthesized in 1-propanol and determined with NMR and aqueous SEC using PEO calibration with $M_{n,theo}$ = 8.5 kDa and a M_w/M_n = 1.09.

Time [min]	Conversion [%]	r_{DLS} [nm]	*PDI*	r_{TEM} [nm]	$M_{n,theo}$ [kDa]	$M_{n,exp}$ [kDa]	M_w/M_n
20	0	-	-	-	-	-	-
40	0	-	-	-	-	-	-
60	2.0			-	10	-	-
80	5.1	50.1 ± 0.5	0.07 ± 0.02	-	12	10.2	2.63
120	22	59.2 ± 0.5	0.04 ± 0.02	39 ± 4	26	41.9	1.27
145	38	62.7 ± 0.5	0.03 ± 0.03	52 ± 6	38	57.7	1.32
160	45	65.3 ± 0.8	0.07 ± 0.02	55 ± 4	43	69.9	1.40
180	60	69.6 ± 0.2	0.1 ± 0.1	60 ± 5	55	91.4	1.43

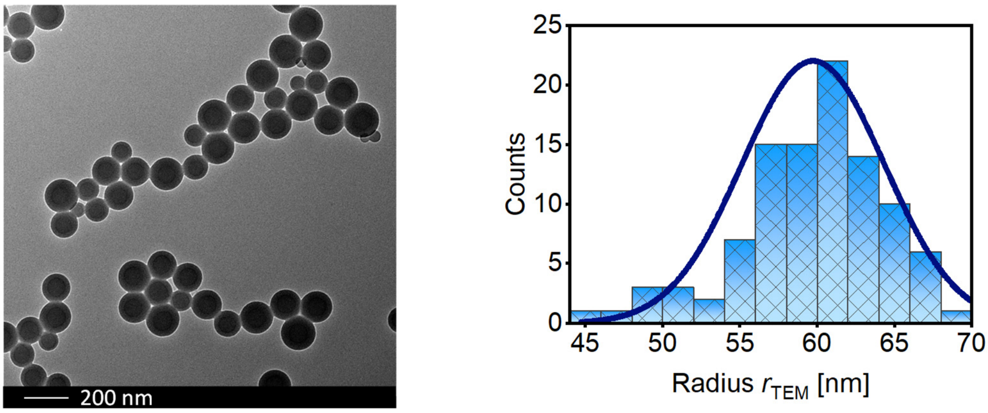

Figure S7. TEM image (left) and histogram (right) of the number-average radius of the of $PMAA_{10}$-*b*-PS_{90}^{86} after 180 minutes reaction time with CTA3 copolymer and 50 wt% and 90 % weight fraction of styrene.

Table S10. Kinetic Data of the Polymerization of $PMAA_5$-*b*-PS_{95}^{165} with CTA3 at 80 °C in water with a targeted styrene DP of 1500 with a [CTA3]/[ACVA] molar ratio of 5 at 50 wt%. r_{TEM} represents the number-average radius obtained by TEM and r_{DLS} by DLS. $M_{n,theo}$ represents the theoretical number-average molecular weight of PMAA-*b*-PS and $M_{n,theo^*}$ the theoretical number-average molecular weight of PMMA-*b*-PS. The dispersity M_w/M_n was determined my SEC THF + 35 mmol TFA using universal PS calibration. The PMAA-CTA3 macro-CTA was synthesized in 1-propanol and determined with NMR and aqueous SEC using PEO calibration with $M_{n,theo}$ = 8.5 kDa and a M_w/M_n = 1.09.

Time [min]	Conversion [%]	r_{DLS} [nm]	*PDI*	r_{TEM} [nm]	$M_{n,theo}$ [kDa]	$M_{n,exp}$ [kDa]	M_w/M_n
20	0	-	-	-	-	-	-
40	0	-	-	-	-	-	-
60	0	-	-	-	-	-	-
80	1	-	-	-	10	-	-
100	3	58.4 ± 0.1	0.07 ± 0.02	-	14	34.1	1.15
120	6.9	61.1 ± 0.7	0.06 ± 0.02	-	19	37.1	1.24
140	8.9	64.6 ± 0.8	0.06 ± 0.05	-	22	43.5	1.28
160	11	66.7 ± 0.5	0.06 ± 0.02	-	26	45.1	1.30
180	13	69.5 ± 0.4	0.06 ± 0.02	55 ± 7	28	45.7	1.40

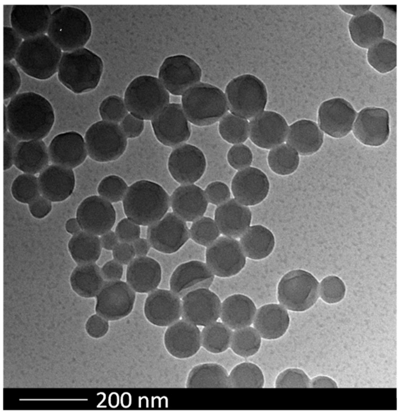

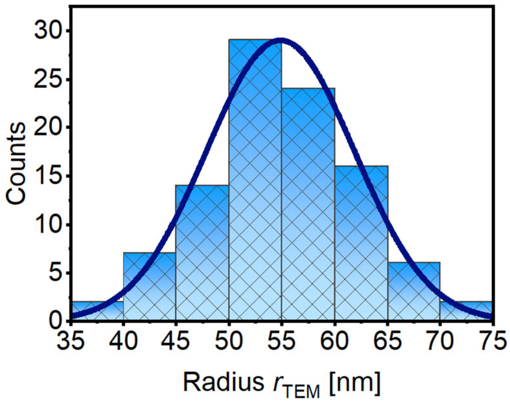

Figure S8. TEM image (left) and histogram (right) of the number-average radius of the of $PMAA_5$-*b*-PS_{95}^{165} after 180 minutes reaction time with CTA3 copolymer and 50 wt% and 90 % weight fraction of styrene.

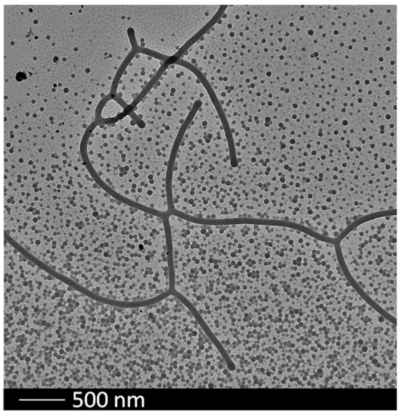

Figure S9. TEM image of $PMAA_{40}$-*b*-PS_{60}^{46} after full conversion with CTA1 copolymer and 20 wt%.

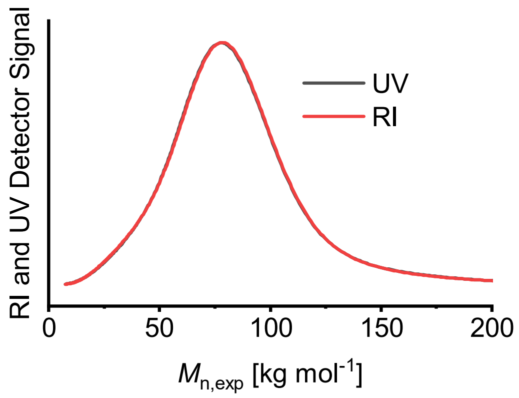

Figure S10. SEC results of $PMAA_{20}$-*b*-PS_{80}^{42} with CTA3 at 50 wt% after full conversion.

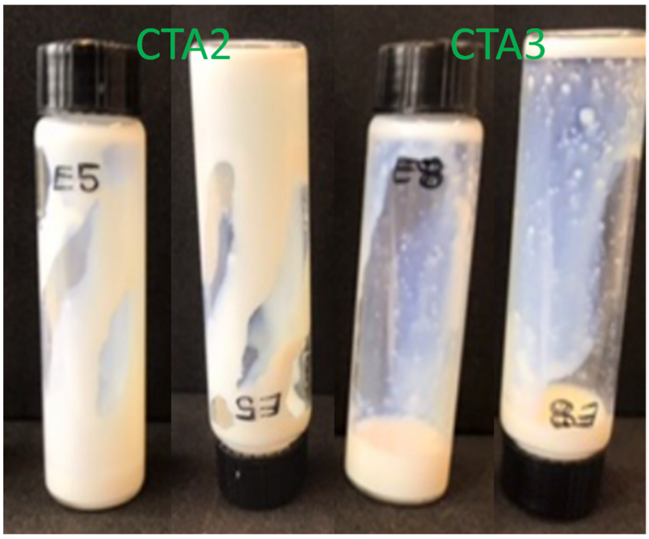

Figure S11. Pictures of the polymerization of PMAA-*b*-PS with CTA2 and CTA3 at 40 wt%. The polymerization with CTA2 shows clearly a more viscous medium.

Table S11. Overview of the PMAA-*b*-PS polymerizations with the corresponding macro-CTAs. SEC data for macro-CTA1 for polymer $PMAA_{10}$-*b*-PS_{90}^{45} was determined with aqueous SEC using PAA calibration. All other macro-CTAs were measured against PEO calibration as it was mentioned in the previous tables. For more detailed information see Table S3-S10.

Targeted polymer	Time [min]	(*w/w*) solids [%]	Macro-CTA	DP PMAA	$M_{n,theo}$ PMAA [kDa]	M_n,M_w PMAA	Targeted DP PS	Targeted PS $M_{n,theo}$ [kDa]	PS Conversion	$M_{n,exp}$ PMAA-*b*-PS [kDa]	M_n,M_w PMAA-*b*-PS
$PMAA_{05}$-*b*-PS_{95}^{54}	120	20	CTA1	30	2.9	1.06	502	52.3	99	69.1	1.33
$PMAA_{10}$-*b*-PS_{90}^{45}	120	20	CTA1	50	4.5	1.25	400	41.7	97	69.5	1.26
$PMAA_{10}$-*b*-PS_{90}^{45}	120	25	CTA1	50	4.5	1.25	400	41.7	94	71.0	1.50
$PMAA_{10}$-*b*-PS_{90}^{45}	300	30	CTA1	50	4.5	1.25	400	41.7	28	26.0	1.38
$PMAA_{20}$-*b*-PS_{80}^{46}	240	35	CTA2	101	8.7	1.17	360	37.4	99	166	1.57
$PMAA_{20}$-*b*-PS_{80}^{46}	240	40	CTA2	101	8.7	1.17	360	37.4	99	158	1.61
$PMAA_{20}$-*b*-PS_{80}^{18}	80	40	CTA3	43	3.8	1.11	140	33.2	91	59.0	1.23
$PMAA_{20}$-*b*-PS_{80}^{18}	80	50	CTA3	43	3.8	1.11	140	33.2	94	63.0	1.23
$PMAA_{20}$-*b*-PS_{80}^{42}	140	50	CTA3	95	8.5	1.09	320	33.2	99	59.6	1.22
$PMAA_{10}$-*b*-PS_{90}^{86}	180	50	CTA3	95	8.5	1.09	740	77.0	60	91.4	1.43
$PMAA_{05}$-*b*-PS_{95}^{165}	180	50	CTA3	95	8.5	1.09	1500	156	13	45.7	1.40
